# Noise annoyance during COVID-19 lockdown: A research of public opinion before and during the pandemica)

**DOI:** 10.1121/10.0002667

**Published:** 2020-12-08

**Authors:** Ayça Şentop Dümen, Konca Şaher

**Affiliations:** 1 Istanbul Bilgi University, Kazım Karabekir Caddesi, 34060, Eyüp, Istanbul, Turkey; 2 Kadir Has University, Kadir Has Caddesi, 34083, Fatih, Istanbul, Turkey

## Abstract

Strict lockdown strategies to stop the spread of COVID-19 have caused a decrease in environmental noise levels and introduced new noise conditions in dwellings. The present study has investigated the impact of the forced lockdown in Turkey on noise annoyances due to traffic, neighbors, and personal dwellings, as well as the concern of being heard by neighbors, and overall dwelling satisfaction in an online questionnaire. The stress and anxiety levels of respondents were also investigated. The survey obtained 1053 respondents. Additionally, environmental noise levels were measured over 24-h at two locations and compared with results before the pandemic. The results clearly exhibit that environmental noise levels and annoyance due to the noise levels dropped significantly. The annoyance drop was larger in previously noisier environments than previously tranquil locations. Noise annoyance due to neighbor noise did not change significantly; however, noise annoyance due to one's own dwelling increased. The results also confirmed an overall increase in dwelling satisfactions indicating a correlation between dwelling satisfaction and lower environmental noise levels. Although the results confirmed that noise annoyance was positively correlated with stress and anxiety levels, the change of annoyance between before and during lockdown was shown to be independent from the stress and anxiety level.

## I. INTRODUCTION

All around the world, governments adopted various strategies and brought strict restrictions to fight against the spread of the COVID-19 pandemic. Restricting people's activities, thus decreasing contact risk, was a common solution, although what one can or cannot do varied greatly among countries (Hirsch, 2020). Most countries applied lockdowns, others banned non-essential movements, and some applied even stricter lockdowns and curfews, where life had stopped except for essential services (e.g., hospitals, police) and people were banned from the streets for some time. International travels were restricted, events were cancelled, and outdoor activities were controlled to some level in almost all of Europe. Comparison between countries' COVID-19 management strategies is possible using the Oxford Government Response Tracker (Oxford University, 2020), which can be useful for the readers to relate the following results to their own experiences. This tool calculates a Government Response Stringency Index between 0 and 100 using nine restriction indicators. To give a general idea, on April 29 (when the research started) Turkey's score was 75.93, China's 56.94, United States' 72.69, Italy's 93.52, and Sweden's 46.3. On May 31 (when the research ended), Turkey's score was still 75.93, China's 81.94, United States' 72.69, Italy's 63.89, and Sweden's 46.3.

The slowed down period had consequences in our environments as well as in noise issues in our dwellings. The most obvious expectance would be a decrease in environmental noise levels. However, several other factors can also be listed to describe the specific sound environment and people's reaction during lockdown. First, the amount of time spent at home increased sharply; thus, the duration of exposure to existing noise sources at one's house (traffic, neighbor, or other types) increased. Second, living in a lockdown induced higher stress and anxiety levels, which may have indirectly affected how people observed their environments. Third, the occupant's activities varied as dwellings transformed into a single compact environment where people live, work, learn, exercise, mediate, interact virtually with friends, and telecommute, while also sharing this space with others and their preferred activities. This meant changes in both neighbor noise properties and task-specific acoustic requirements. An architect anticipated that “Acoustic divisions have become more important while the family is crammed in together all day long. The loft, the New York City typology, seems to be not the romantic thing at the moment. Everyone's on Zoom calls” (Chayka, 2020). On the other hand, the conclusion of the same article included some hints of opposing the complete isolation, stating the requirement for a “hideaway” as being “full of reminders that the rest of the world still exists.”

Indeed, it is unknown how this experience will shape our future expectations from our dwellings. However, some of these changes will seemingly become permanent in our lives. Experiences with online education encourage new methods and an increase in blended teaching. Working from home brought advantages of cost saving, space use, avoiding rush hour, motivation, and efficiency. Several companies such as Twitter, Barclays, and PSA have already announced that they would continue to implement remote working, flexible hours, and shifts at workplaces. It is important to learn from the experiences of the lockdown period and reshape future requirements to aim for a higher quality in dwellings. With this intention, this paper introduces a national experience from Turkey, aiming to explore the public opinion on noise annoyance specifically in dwellings before and during the lockdown period. The paper also aims to compare the measured environmental noise levels in two buildings before and during the lockdown and explore the impact of induced stress and anxiety levels during the lockdown period on noise annoyance.

## II. OVERVIEW OF THE LOCKDOWN PERIOD

### A. COVID-19 management strategies in Turkey

Compared to other countries, Turkey took a slightly different strategy in fighting the pandemic.

The Turkish Government imposed a partial lockdown where most businesses were open, and total curfews during weekends and holidays. Restrictions were imposed starting from March 15 until June 1, with closure of entertainment facilities, shopping malls, restaurants, parks, beaches and recreational areas. A curfew was declared for the young, elderly, and chronically ill. Education continued online. Some employees went on working remotely and some had to take paid/unpaid leave, which reduced road traffic. Due to low passenger-numbers, Istanbul and Ankara Municipalities reduced the public transport services. Between April 11 and June 1, thirty-one cities were subjected to a total curfew during weekends and public holidays, except for employees with special permission such as home-delivery services, healthcare professionals, security officers, and service personnel. This period of strict restrictions also included the holy Ramadan month and the following three-day festivities, which is a national holiday. Typically, this month and the following holiday period are vibrant times due to family gatherings and collective dinners after fasting and during the festivities; however, curfew in 81 cities during the holiday restricted the activities of people. On the other hand, mosques, which were closed to congregations and collective prayers due to the pandemic, broadcasted prayers and sermons through speakers during this month.

### B. Mental health, COVID-19, and noise annoyance

During the pandemic period, several stressors such as risk of getting infected, self-isolation, job loss, and uncertainties, together with experienced fear, worry, and feelings of powerlessness were threatening the mental health of the public. The Inter-Agency Standing Committee (2020) published a briefing that consisted of global recommendations and warnings regarding the stressors and threats, possible reactions of people, mental health, and the need for psychosocial support. The World Health Organization (2020) promoted messages on the importance of supporting mental and psychosocial wellbeing. Many other scientific studies showed increased levels of anxiety, stress, depression, sleep disorder, fear, and obsessive compulsive disorder (Vindegaard and Benros, 2020). An Increased risk of symptoms was associated with several factors including job related factors (front-line health care workers, people who stopped working), gender (women), and medical history (poor health, chronic disease).

In noise annoyance literature, there are various studies addressing the relation between the mental health of the respondent and the noise annoyance in dwellings. Grøtvedt (1990) stated that people who reported psychiatric problems/diseases suffered more from neighbor noise annoyance. Persson *et al.* (2007) found positive association between the continuous trait anxiety score and annoyances due to traffic, neighbors, ventilation, and related installation annoyances. Van den Berg *et al.* (2014) stated that the risk of suffering from anxiety and depression was associated with annoyance and sleep disturbance. Jensen *et al.* (2018) showed the positive correlation between mental health score and neighbor noise annoyance. Considering the findings in the literature, it can be suggested that people's arguments about noise issues changed parallel to their stress and anxiety caused by lockdown.

### C. Research objectives and the timeline

**FIG. 1.**
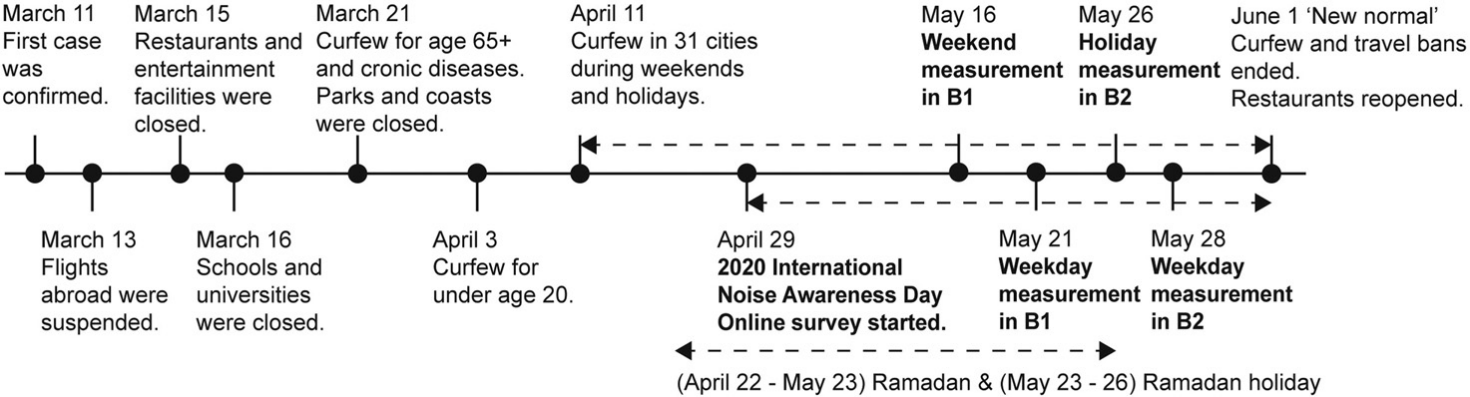
Description of COVID-19 restrictions in Turkey in relation to the research timeline.

The COVID-19 management strategies of Turkey caused unsteady sound environments and sharp differences between before and after COVID-19, partial-lockdown during weekdays, curfew during weekends and holidays, and Ramadan days laying an unprecedented emphasis on noise in our daily lives. This lockdown and curfew periods presented a perfect opportunity to assess the noise perception of people before and during the lockdown and increase awareness on the subject. With this intention, the Turkish Acoustical Society initiated a public opinion survey on International Noise Awareness Day 2020, also contributing to the International Year of Sound activities. The present research aims to investigate the noise issues and perception in dwellings before and during lockdown and gather general public opinion of noise environments using questionnaires. Considering the extreme stressors of the pandemic, the stress and anxiety levels of respondents were also addressed in the questionnaire. The research further aims to compare the environmental noise levels in two buildings with distinct locational characteristics (indicated as B1 and B2), where measurements were taken before and during the lockdown period in order to assess the correlations between the measured noise levels and subjective responses. The timeline of this research in relation to curfews and restrictions are given in Fig. 1.

## III. ENVIRONMENTAL NOISE LEVELS DURING THE LOCKDOWN

### A. Materials and methods

In May 2017, environmental noise levels in some residential buildings were measured for research about the applicability of regulatory criteria for a sound insulation performance assessment in Turkey (Dümen and Bayazıt, 2020). Two residential buildings from this former study were chosen to investigate the environmental noise levels before and during the pandemic. These two buildings were chosen in an effort to represent different types of residential areas in terms of noise levels. Both buildings were located in Istanbul. B1 had high environmental noise levels and was situated close to a main road. B2 had moderate environmental noise levels and was situated in a housing estate surrounded by greenery. Environmental noise levels at these two locations were measured during the partial lockdown and curfew periods to obtain comparable data that may show the changes in environmental noise levels.

The environmental noise levels were measured according to ISO 1996-2 (2017). The distance between the measurement position and the facade was less than two meters; therefore −3 dB correction was applied to account for the facade reflections according to the standard. The 24-h measurements were repeated during (a) total curfew during weekend and holiday and (b) partial lockdown during weekdays.

### B. Comparison of measurement results

The measurement results show that during the regular weekdays of partial lockdown the environmental noise levels were decreased by 2.1 dB at the B1 location (which is near a main road) and 2.4 dB at the B2 location (which is surrounded by greenery) (Table I). It was observed that the balconies were occupied more frequently compared to 2017 measurements in both locations. The total curfew caused a more prominent effect in B1 location with a drop of 7.8 dB. On the other hand, measurement at the B2 location during the curfew was strongly affected by the human activities in the surrounding greenery showing a 5.0 dB increase compared to 2017 measurements. When the logged measurement results were analyzed, a sharp drop from 65 to 54 dB was observed at 11:00 p.m.; implying the end of social activities. A reason for the increased activity was the Ramadan holiday. Since parks and recreational areas were closed during the pandemic, the greenery zones around the apartments and in the housing estates served as a gathering place and activity space for some families in spite of the curfew. Therefore, this measurement accented a transition of environmental noise source from traffic to neighbor activities, seemingly causing even higher noise levels. Although this result may be specific to Ramadan holiday, it is worth questioning the change in balances between residential areas and in the role of greenery zones that were once associated with silence and became a noise source during the lockdown. It will be interesting to see the future implications of the new living-styles and activities in this context. The measurement results provided insight into the current situation; however, they should not be generalized without further evidence.

**TABLE I.** Environmental noise levels comparison.

	L_day_	L_evening_	L_night_	L_den_	L_den_ – 3 dB	Difference
B1—2017 results	73.6	72.3	68.5	76.5	73.5	
B1—total curfew	62.1	59.2	62.6	68.6	65.6	–7.8 dB
B1—partial lockdown	71.9	68.6	66.7	74.3	71.3	–2.1 dB
B2—2017 results	60.1	59.7	58.2	65.1	62.1	
B2—total curfew	71.6	65.8	54.8	70.1	67.1	+5.0 dB
B2—partial lockdown	58.8	59.5	54.8	62.7	59.7	–2.4 dB

## IV. NOISE ANNOYANCE DURING THE LOCKDOWN

### A. Materials and methods

On International Noise Awareness Day 2020, an online public opinion survey was initiated in order to investigate the noise related annoyance in dwellings and to create awareness on the subject.

#### 1. The survey structure

The survey was kept brief to encourage participation, introducing some aspects of noise issues and a comment field where people could describe their additional concerns. The survey structure, questions, and response scales are given in Table II. Annoyance questions were formulated according to ISO/TS 15666 (2003). Annoyances due to (a) traffic noise, (b) neighbor noise, and (c) noise from one's own dwelling were addressed in this questionnaire. In order to account for the two-way transfer of sound, an additional question was introduced about one's “concern of being heard” by his/her neighbors. Finally, “dwelling satisfaction considering the noise” was asked to gain insight into the overall perception of the residents.

**TABLE II.** Survey structure, questions and response scale.

	Question	Response scale
Section 1: “Considering the past one year before COVID-19 pandemic”	Q1. How do you describe the noise level where you live?	0–10 numeric scale with verbal labels only at extreme ends:
	Q2. How much were you annoyed;	Q1. 0 = Very silent, 10 = Very noisy
	… due to traffic noise?	Q2. 0 = Not at all annoyed, 10 = Extremely annoyed
	… due to neighbor noise?	Q3. 0 = Not at all concerned, 10 = Extremely concerned
	… due to noise coming from other rooms inside your dwelling?	Q4. 0 = Not at all satisfied, 10 = Very satisfied (The extreme end of the positive response scale was labeled with “very” instead of “extremely” due to the negative association of the word in Turkish.)
	Q3. How much were you concerned of your own noise getting heard by your neighbors?	
	Q4. How much are you satisfied with your dwelling considering the noise issues?	
Section 2: “Considering the past one month during COVID-19 pandemic”	Q5–Q8. Same as section 1.	Q5–Q8. Same as section 1.
Section 3: General situation	Q9. In general, how sensitive are you to noise?	Q9. 0–10 numeric scale with verbal labels only at extreme-ends: 0 = Not at all sensitive, 10 = Very sensitive
	Q10. Short version of Perceived Stress Scale (PSS-4)	Q10. 5 point verbal scale: 1-Never / 2-Almost never / 3-Sometimes / 4-Fairly often / 5-Very often
	Q11. Short version of State—Trait Anxiety Inventory (STAI-6)	Q11. 4 point verbal scale: 1-Not at all / 2-Somewhat / 3-Moderately / 4-Very much
	Q12. How frequently did you leave your house during the last month?	Q12. Almost never / Once in every two days / Almost every day
	Q13. How frequently did you spend time on works requiring concentration such as working from home, writing, reading during the last month?	Q13. Never / Seldom / Couple of hours in a week / Less than two hours on a day / More than two hours on a day
	Q14. How can you describe the building you are living in?	Q14. Single family house / Apartment with 2–7 floors / Apartment with 8–18 floors / Apartment with more than 18 floors
	Q15. In which city is the building located?	Q15, Q18, Q19. Open-ended
	Q16. Gender, Q17. Age, Q18. Occupation, Q19. Comments	

In order to measure the stress level, the Perceived Stress Scale (PSS) was used in its short form. The original PSS inventory involved fourteen questions, but studies showed that the shorter version (PSS-4), which involved four questions, was also reliable (Cohen *et al.*, 1983). The questions are related to how uncontrollable, unpredictable, or overloaded life is as perceived by the respondent. Application of PSS inventory in annoyance research was also used by Jensen *et al.* (2018). The Turkish translation of the inventory was derived from Eskin *et al.* (2013), where the reliability of long and short forms in Turkish was shown to be sufficient. The anxiety level was measured with the short version of the Spielberger State–Trait Anxiety Inventory (STAI-6). The original inventory included forty questions (Spielberger *et al.*, 1983); however, short forms were developed, and reliabilities were shown in time (Marteau and Bekker, 1992; Tluczek *et al.*, 2009). The Inventory was translated to Turkish and found use in some medical studies (Oner and Le Compte, 1983).

#### 2. Data collection

The online survey was started at the Turkish Acoustical Society's webpage, announced to the public during the broadcast of International Noise Awareness Day, in social media, in the society's newsletter, and through networks of board members. The survey took approximately eight minutes to answer. Personal information was not collected during the survey. A total of 1053 responses were obtained. Since the survey addressed how the noise annoyance was affected corresponding to changes in noise levels, responses of four people who were relocated during the pandemic were eliminated. Another potential bias would be related to our online survey method that did not include IP tracking or sign-in options to encourage participation. This could result in “double-clicking” on the approval screen and might damage the data quality. To avoid this, the exact same answers were detected by comparing all given answers including the individual answers to the open-ended questions in a two minutes frame. Seven responses were eliminated for this reason. Twenty-one responses from residents in other countries were also not analyzed. As a result, 1021 responses were evaluated in this study.

#### 3. Analysis of the results

The results were analyzed with IBM SPSS statistics software v.20. Statistical significance of differences between evaluations of two time periods (before and during the lockdown) was tested.

The difference between the paired values showed normal distribution, and a paired samples t test was applied. Second, statistical significance of differences between evaluations of two independent groups was tested (group-mean equality test). Groups were formed according to (1) environmental noise level evaluation, (2) stress score, (3) anxiety score. Environmental noise level evaluations smaller than 3/10 and higher than 7/10 were grouped and named “lower noise level evaluation” and “higher noise level evaluation.” Each item of PSS-4 and STAI-6 were summed separately and the results were shown as overall stress and anxiety scores over 100. For group comparison, cut points were determined according to the 30% of the population that had the lowest and the highest scores. Resultant cut points were 45 and 65 for the PSS-4 score and 50 and 71 for the STAI-6 score. Thus, two equal sized groups were created and named “low-stress” and “high-stress” or “low-anxiety” and “high-anxiety.” An unpaired (independent) samples t test was applied to test the group differences. The mean evaluations and *p*-values are given in charts and compared to a 5% statistical significance level. Finally, Pearson's correlation test was applied to measure the associations between parameters.

### B. Survey results

Seventy-one of the respondents lived alone and they did not evaluate noise from their own dwelling. Fifty-three people left explanations and comments regarding sounds and annoyance. The sample had a balanced age distribution representing the population (16–25: 20%, 25–35: 24%, 35–45: 22%, 45–55: 17%, 55–65: 16%, 65+: 4%); however, men were under-represented (30%). The dataset was weighted with respect to gender to represent equal distribution among men and women. Annoyances due to (1) traffic noise, (2) neighbor noise, (3) noise from one's own dwelling, (3) concern of being heard, and (4) dwelling satisfaction were dealt as response parameters and used for analyzing and reporting results.

#### 1. House use during the lockdown

**FIG. 2.**
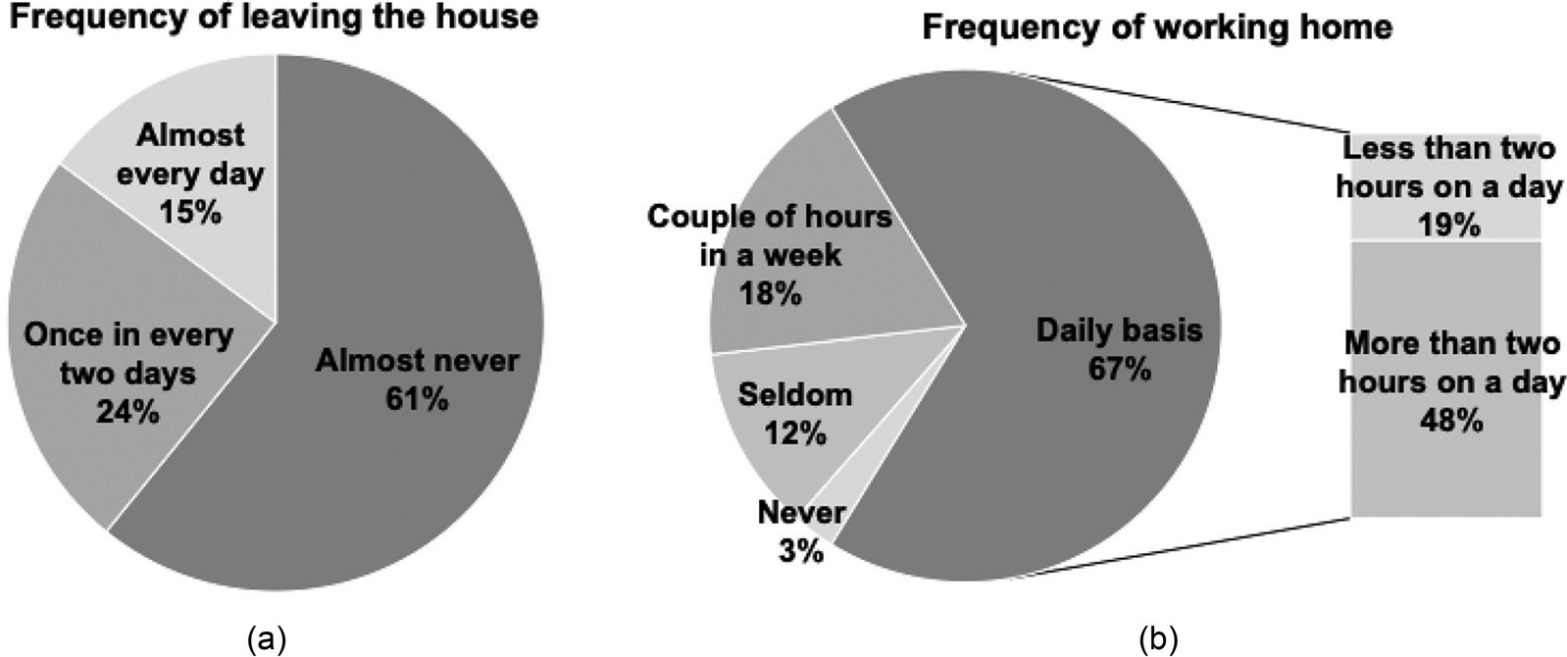
Indicators of house use during the lockdown period. Distribution of results according to (a) frequency of leaving the house, (b) frequency of working home.

The definition of “dwelling” has changed significantly during the lockdown. The survey shows that 61% of the respondents did not leave the house during the pandemic; while 67% reported that on a daily basis they dealt with work that required concentration in their houses (Fig. 2). These social changes are expected to have a permanent effect on our lives even after the pandemic. Corresponding to all of the new functions that our dwellings have earned, the sound insulation demand may also increase, therefore providing an important aspect.

#### 2. Changes between two time periods

As expected, environmental noise level evaluation and annoyance due to traffic noise was significantly decreased during the lockdown, while annoyance due to noise from one's own dwelling increased. Annoyance due to neighbor noise did not change significantly even after single-family houses were removed from the database. This is interesting because an increased annoyance would be expected by respondents' comments and by the fact that people spent more time in their homes during the pandemic increasing noise durations and exposure. Table III also shows that the mean values of all noise parameters investigated increase when single-family houses (detached or semi-detached) are removed from the assessment, therefore, stressing the effect of typology of dwellings.

**TABLE III.** Comparison of mean evaluations before and during the lockdown.

		Mean noise annoyance			Mean concern of being heard	Mean environ. noise level evaluation	Mean dwelling satisfaction
		Traffic	Neighbor	Dwelling			
All responses (n = 1021)	Before	3.79	4.12	2.68	5.20	4.87	6.11
	During	2.67	4.13	2.94	5.11	3.75	6.36
	t	15.22	−0.35	−0.54	1.62	15.22	−3.67
	p	<0.001	0.729	<0.001	0.106	<0.001	<0.001
Without single-family houses (n = 865)	Before	4.01	4.31	2.78	5.41	5.14	5.92
	During	2.84	4.38	3.09	5.34	3.91	6.21
	t	14.55	−1.12	−6.03	1.20	15.23	−4.13
	p	<0.001	0.262	<0.001	0.232	<0.001	<0.001

In order to show the exact change between two time periods, the mean “annoyance,” “concern of being heard,” “sound level,” and “dwelling satisfaction” evaluations regarding the past year were subtracted from the mean evaluations regarding the current situation (E_COVID-19_ – E_year_). As observed in the measurement results there may be differences in noise characteristics between different noise zones. Since the environmental noise exposure of respondents was unknown, the responses were grouped according to environmental noise level evaluations before the pandemic. However, it should be noted that responses to this question depends not only on noise levels but also on several other factors such as noise sensitivity and time spent at home.

Evaluations smaller than 3/10 provided basis for “lower noise level evaluation” and evaluations higher than 7/10 provided basis for “higher noise level evaluation.” It is shown in Table IV that the changes in traffic noise annoyance, environmental noise level evaluations and dwelling satisfaction are sharper in areas evaluated with higher noise levels.

**TABLE IV.** Changes between evaluations regarding the two time periods (nr: not relevant).

	Change in noise annoyance			Change in concern of being heard	Change in environ. noise level evaluation	Change in dwelling satisfaction
	Traffic	Neighbor	Dwelling			
Overall (n = 1021)	−1.12	+0.02	+0.25	−0.09	−1.12	+0.25
Lower noise level evaluation (n = 327)	−0.32	nr	nr	nr	−0.46	−0.99
Higher noise level evaluation (n = 305)	−2.00	nr	nr	nr	−2.33	+0.72

There were fifty-three comments from participants that gave insight into other dimensions of the subject. Some addressed their positive feelings due to presence of sounds; birds (4), neighbors (1); or absence of sounds; traffic and horn (7), people outside (3), outside noises in general (3). Some wanted to express their annoyance due to neighbor activities [in general (6), children crying (3), music (3), cleaning (1), flushing (1), telephone speech (1), balcony activities (1)], due to environmental noise [mosque (5), people outside (3), construction (3), car alarms (1)], due to noise from their own dwelling [children (1), TV (1)], and due to concern of being heard (3). Twelve comments stated an increased awareness of sound environment related with being more conscious of noise sources than before or “discovering”/“realizing” some sounds. Another seven stressed the need for awareness in society and their expectation of a solution for noise issues. Finally, seven people blamed their buildings while eight people blamed their neighbors for their exposure to noise.

#### 3. Stress, anxiety, and annoyance

Among fifty-three comments left in the survey, ten were related to sounds effects on their wellbeing. While five of them claimed positive sounds and absence of noise decreased their stress and anxiety, three of them mentioned that their stress and anxiety increased due to noises in lockdown period.

**FIG. 3.**
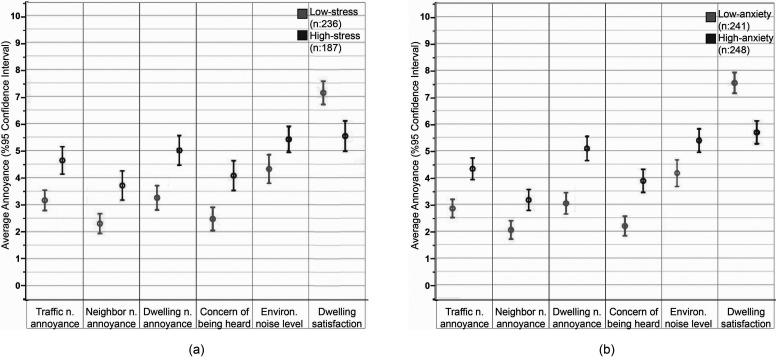
Mean values of annoyance, concern, noise level, and satisfaction ratings during the COVID-19 period, according to (a) stress level and (b) anxiety level.

A correlation analysis showed that noise sensitivity, stress score, and anxiety score were all positively correlated with the response parameters: annoyance, concern of being heard, satisfaction (Table V). Noise sensitivity was correlated with stress score [r(1019) = 0.092, p = 0.003] and anxiety score [r(1019) = 0.101, p = 0.001]. Stress and anxiety scores were negatively correlated with dwelling satisfaction while correlation between noise sensitivity and dwelling satisfaction was insignificant in lockdown period. This indicates several other factors were effective in this judgment. The results were further analyzed by grouping the responses that are in the 30% slices of the two tail ends of the distribution. Figure 3 shows the mean evaluations for these groups regarding the COVID-19 period. Results confirmed that stress/anxiety levels are directly correlated to noise annoyance and the other parameters of this study.

**TABLE V.** Correlation analysis of stress/anxiety and noise annoyance parameters (r: Pearson correlation coefficient, p: significance, **correlation at the 0.01 level).

		Evaluations regarding one year period (before pandemic)						Evaluations regarding the COVID-19 period					
		Noise annoyance			Concern of being heard	Env. noise level ev.	Dwelling Satisfaction	Mean noise annoyance			Concern of being heard	Env. noise level ev.	Dwelling satisfaction
		Traffic	Neighb.	Dwelling				Traffic	Neighb.	Dwelling			
Noise Sensitivity	r	0.099**	0.208**	0.142**	0.162**	0.137**	−0.123**	0.109**	0.185**	0.135**	0.138**	0.155**	−0.044
	p	0.002	<0.001	<0.001	<0.001	<0.001	<0.001	<0.001	<0.001	<0.001	<0.001	<0.001	0.156
Stress score	r	0.118**	0.169**	0.138**	0.115**	0.156**	−0.153**	0.121**	0.167**	0.144**	0.105**	0.154**	−0.155**
	p	<0.001	<0.001	<0.001	0.001	<0.001	<0.001	<0.001	<0.001	<0.001	0.003	<0.001	<0.001
Anxiety score	r	0.111**	0.195**	0.195**	0.125**	0.154**	−0.206**	0.129**	0.206**	0.216**	0.145**	0.209**	−0.209**
	p	<0.001	<0.001	<0.001	0.001	<0.001	<0.001	<0.001	<0.001	<0.001	<0.001	<0.001	<0.001

It was also investigated whether stress and anxiety determined the amount of change in respondents' evaluation of noise annoyance between the two periods. One hypothesis can be that people who expressed more changes in their evaluations of these two periods can also have higher stress levels. The group comparison test was repeated to assess the degree of changes between two time periods (E_COVID-19_ – E_year_). The high stress/anxiety group experienced somewhat larger changes in annoyance due to traffic noise, annoyance due to their own dwelling noise, and in the perceived environmental noise level, however, the increase in dwelling satisfaction was less compared to the low stress/anxiety group. Neighbor noise annoyance and concern of being heard changed in the opposite directions for the two groups; it increased for the high-stress/anxiety group and decreased for the low-stress/anxiety group. However, the group differences failed the statistical importance test. The low correlation confirmed that changes in respondents' evaluation of noise annoyance were attributable only to changes in noise conditions.

## V. CONCLUSION AND THE FUTURE AGENDA

In order to investigate the effect of lockdowns on perception and create awareness, this research included a public opinion survey on noise annoyance and some environmental noise measurements. The measurement results showed a decrease of 2.2 dB during the partial lockdown. During the curfew, a decrease of 7.8 dB was observed near a main road and an increase of 5 dB was noted in a housing estate due to people's outdoor activities. A larger amount of measurements would be necessary to generalize the results, however, the study faced challenges brought by strict lockdown measures. Nevertheless, the results provided objective data on the situation and supported survey findings on perceived drop in environmental noise levels and different characteristics of areas evaluated with higher and lower noise levels. The survey results showed that in noisier locations people's annoyance and noise level evaluations changed more sharply than tranquil locations during the pandemic. Annoyance due to traffic noise was decreased as expected; annoyance due to noise from one's own dwelling was increased, neighbor noise annoyance, on the other hand, did not change significantly. The dwelling satisfaction considering the noise in general increased, showing that people considered more environmental noise while responding to this question. Stress and anxiety levels were found to be associated with the noise sensitivity and annoyance findings, confirming former studies in the literature. However, the change of annoyance between two time periods (E_COVID-19_ – E_year_) was independent from stress and anxiety levels, which means that both high stress/anxiety and low stress/anxiety groups were affected similarly by the changing noise conditions. Out of 53 written comments 19 (%36) mentioned “noise awareness”: 12 (%23) stated that their noise awareness increased during the pandemic and another 7 (%13) stressed the need for awareness in society.

COVID-19 lockdowns came as a shock for most city people, who had become desensitized by the pace of life, crowds, being exposed to all kinds of contents, and using their dwellings mainly for sleeping. But in the dystopia of COVID-19, the relation of people with their living environment has sharpened. Some found peace by hearing the birds again, some got upset by their neighbors' music or cats, one person felt less alone when hearing their neighbors, and a student was less content with distractions during online education. Whether their experience was positive or negative, the lockdown seemingly reminded people of the importance of their living environment, increasing their awareness and reshaping expectations. In the post-pandemic era, there will be time to discuss how to improve the quality of dwellings in relation to comfort and wellbeing. Architects are now searching for new and flexible solutions that will answer the needs for various inclusive or private activities. Acoustical engineers should guide them in designing and detailing better sound insulation to meet the requirements, which will be redefined considering the changes in noise characteristics, exposure durations, and occupant activities. In the near future, people will probably consider how it would be like to shelter in place when they need to decide on a house. Several aspects such as spaciousness or daylight will be obvious selection criteria. However, despite being an important criterion, they will not know the sound insulation before they move-in or until the next lockdown when the neighbors will increase their sound. This highlights the importance of improved regulations, acoustic classification, and labeling through measurements. Sound insulation of both exterior and interior building elements in dwellings should be considered and studies and events should be planned to increase public awareness.
